# Psychological and lifestyle correlates of eating behavior and adiposity: Structural and latent profile modeling

**DOI:** 10.1371/journal.pone.0343336

**Published:** 2026-02-20

**Authors:** Małgorzata Obara-Gołębiowska

**Affiliations:** Department of Clinical, Developmental and Educational Psychology, Faculty of Social Sciences, University of Warmia and Mazury in Olsztyn, Olsztyn, Poland; University of Technology Sydney, AUSTRALIA

## Abstract

**Background:**

Psychological vulnerabilities, including early maladaptive schemas (EMS) and difficulties in emotion regulation, are associated with dysregulated eating and adiposity. However, evidence integrating these mechanisms with contextual and lifestyle factors within a single framework remains limited. This study examined an integrative model of psychological and lifestyle correlates of eating behavior and adiposity in a large adult sample.

**Methods:**

A community sample of 1,500 adults (53% women; aged 18–65 years) completed validated measures of EMS, difficulties in emotion regulation, perceived stress, social support, eating behaviors, diet quality, and physical activity. Body mass index and waist circumference were assessed using standardized procedures. Structural equation modeling tested direct, indirect, and conditional associations, with multi-group analyses examining gender and age differences. Latent profile analysis identified subgroups with distinct psychological and lifestyle constellations.

**Results:**

Higher EMS were associated with greater difficulties in emotion regulation. Emotion regulation difficulties were positively associated with emotional and habitual overeating (stronger among women) and showed a modest negative association with dietary restraint. Indirect effects of EMS via emotion regulation were small and limited to dietary restraint. Perceived stress did not moderate the EMS–emotion regulation association, whereas perceived social support showed a small buffering effect. Eating behaviors were associated with poorer diet quality related to higher body mass index and waist circumference, while physical activity and sedentary behavior showed independent associations with adiposity. Latent profile analysis supported a two-profile solution (higher- vs. lower-risk), characterized by distinct psychological, behavioral, and adiposity patterns.

**Conclusions:**

Cognitive–emotional vulnerabilities are associated with eating dysregulation and adiposity, but emotion regulation plays a selective and modest mediating role limited to dietary restraint. Lifestyle behaviors contribute independently to adiposity alongside psychological pathways, supporting integrative, multidimensional models. Given the cross-sectional design, all findings are correlational and do not imply causality.

## Introduction

### Early maladaptive schemas and eating behavior

Early maladaptive schemas (EMS) are enduring cognitive–emotional patterns that emerge during childhood in response to unmet emotional needs and adverse interpersonal experiences. They represent maladaptive themes about the self, others, and relationships, and they strongly influence affect regulation and coping behaviors [1,2]. Prior research has linked EMS to a range of psychological difficulties, including depression, anxiety, and personality disorders [1], but there is growing recognition that EMS are also associated with health-related behaviors such as eating [3,4].

Evidence suggests that EMS are associated with overeating and weight-related difficulties through their impact on self-regulation and stress responsiveness [3,4]. For instance, schemas in the Disconnection/Rejection domain (e.g., Emotional Deprivation, Abandonment) have been linked to heightened emotional vulnerability [2], while schemas in the Impaired Limits domain (e.g., Insufficient Self-Control) have been associated with difficulties in self-regulation that may predispose individuals to impulsive eating and challenges adhering to dietary goals. In community-based studies, individuals with overweight and obesity endorse higher levels of these EMS compared to normal-weight participants, with women and younger adults in particular reporting stronger schema endorsement and more emotional overeating [5]. These findings highlight EMS as transdiagnostic cognitive–emotional structures that may shape vulnerability to dysregulated eating.

### Emotion regulation and disordered eating

Emotion regulation (ER) difficulties are consistently implicated in maladaptive eating patterns [6]. ER refers to the ability to identify, understand, and modulate emotional states in adaptive ways. Individuals experiencing ER difficulties often struggle with heightened negative affect and may turn to food as a coping strategy [7]. Emotional overeating, in particular, has been described as an attempt to downregulate distress through food consumption, despite its long-term negative consequences for weight and health [8].

Studies demonstrate that ER difficulties are associated with emotional and habitual overeating [9] and may also mediate associations between EMS and disordered eating attitudes [10]. Evidence from both clinical and community samples suggests that ER difficulties are robust correlates of a range of problematic eating behaviors [11]. Longitudinal findings further show that ER difficulties are prospectively linked to weight gain and poorer dietary quality [12,13], suggesting that ER may represent a key psychological mechanism through which broader cognitive–emotional vulnerabilities relate to eating behavior over time [14,15].

### Stress and social support as contextual factors

Beyond cognitive–emotional vulnerabilities, contextual factors such as perceived stress and social support play meaningful roles in shaping eating behavior. Stress is a well-established correlate of overeating, particularly of calorie-dense, palatable foods. Experimental and observational studies have consistently reported that stress is associated with increased emotional eating and reduced dietary restraint [16–18]. Stress has also been linked to both greater ER difficulties and stronger activation of EMS, indicating that it may exacerbate existing psychological vulnerabilities.

Conversely, perceived social support functions as a protective factor, mitigating the extent to which psychological vulnerabilities relate to eating behavior. Social support has been shown to buffer stress and reduce reliance on maladaptive coping strategies such as overeating [19]. In community-based samples, higher perceived social support has been linked to healthier dietary patterns, with evidence that these associations may involve self-efficacy and body-related concerns [20]. Notably, social support appears to operate primarily at the level of eating behavior rather than adiposity itself, highlighting its role as a contextual resource that facilitates adaptive coping.

Taken together, these findings suggest that stress and social support may act as moderators of the pathways linking EMS, ER, and maladaptive eating. Stress may strengthen associations between EMS and ER difficulties, whereas social support may attenuate them. Understanding these moderating effects is essential for developing a nuanced account of how psychological and contextual factors jointly relate to eating behavior.

### Lifestyle factors: Diet quality and physical activity

Lifestyle behaviors such as diet quality and physical activity represent additional factors relevant to understanding eating and adiposity. Psychological studies often rely on self-report measures that capture tendencies such as emotional or habitual overeating; however, such instruments do not directly reflect actual food consumption [21]. Nutritional epidemiology therefore emphasizes validated dietary assessment tools such as food frequency questionnaires (FFQ), which allow quantification of habitual dietary intake [22]. In Poland, the FFQ-6 has been validated and is widely used to assess both healthy and unhealthy eating patterns, including the Unhealthy Diet Index (UDI) [23]. Higher UDI scores have consistently been associated with obesity risk and adverse health outcomes [24]. Moreover, recent research highlights links between consumption of ultra-processed foods, obesity, and psychological distress, including emotional eating [25,26].

Physical activity constitutes another lifestyle factor with implications for both physiological and psychological functioning. Insufficient physical activity and prolonged sedentary behavior often co-occur with unhealthy dietary habits, amplifying both health-related and emotional risks [27]. The International Physical Activity Questionnaire (IPAQ) has demonstrated reliability and validity across diverse cultural contexts [25], and standardized guidelines exist for its scoring and interpretation [28]. Despite its relevance, physical activity is rarely examined alongside deeper cognitive–emotional factors such as EMS and ER within a unified analytical model.

### Why structural equation modeling and latent profile analysis

Although the psychological correlates of eating behavior have been extensively examined using group-comparison and regression-based approaches, much of this work has focused on isolated constructs or specific pathways rather than on their joint organization within a broader system. As a result, less is known about how cognitive–emotional vulnerabilities, contextual factors, and lifestyle behaviors co-occur and integrate in relation to adiposity outcomes in community samples. Although EMS, ER difficulties, perceived stress, and social support have each been individually linked to eating behaviors and adiposity, prior research has typically examined these constructs separately rather than within integrative analytical frameworks. Regression-based approaches have provided valuable insights into direct associations but remain limited in their ability to capture complex mediating and moderating mechanisms among psychological and lifestyle determinants of weight [5]. Structural equation modeling (SEM) offers a comprehensive framework to simultaneously estimate multiple pathways, incorporate latent constructs, and account for measurement error [29–31]. SEM is therefore well suited for testing integrative models in which EMS are associated with eating behaviors indirectly via ER difficulties, with stress and social support acting as contextual moderators. Moreover, SEM allows for multi-group analyses to examine whether these pathways differ by gender and age.

Importantly, despite the theoretical relevance of EMS, ER, stress, social support, diet quality, physical activity, and adiposity, no previous study has integrated all these constructs within a single SEM framework in a large adult sample. This gap underscores the novelty of the present study’s approach.

Complementing SEM, latent profile analysis (LPA) enables the identification of subgroups of individuals who share similar psychological and behavioral characteristics. Prior work in lifestyle and nutrition research has often revealed two or three latent classes [32,33], typically reflecting gradations of risk (e.g., low-, intermediate-, and high-risk profiles). However, the exact number of meaningful psychological–behavioral profiles is empirically determined and may vary across samples. Extending LPA to the psychological domain provides an ecologically valid way to integrate EMS, ER difficulties, stress, and social support with lifestyle indicators. Prior research indicates that individuals high in emotional vulnerability and low in emotion regulation tend to report more overeating and weight-related problems [3,14,15], whereas individuals with higher social support tend to show healthier eating patterns [19]. Women and younger adults also tend to endorse more maladaptive schemas and report more emotional eating [5].

Together, these findings justify the expectation that multiple latent profiles reflecting distinct constellations of psychological and lifestyle factors may be identified, although their number and structure remain exploratory.

### Integrative conceptual model

Eating behaviors result from a complex interplay of cognitive, emotional, and social factors. EMS, conceptualized as pervasive cognitive–emotional patterns developed in childhood, may relate to emotion regulation difficulties [1,7,34,35], which in turn are associated with maladaptive coping strategies such as emotional or habitual overeating. Perceived stress reflects the extent to which individuals appraise life situations as unpredictable or overwhelming [36]. In line with the transactional model of stress and coping [37] and classic buffering models of social support [38], higher stress may coincide with greater ER difficulties, whereas social support may buffer these associations.

Given the cross-sectional design, all pathways in the model were conceptualized as associations rather than causal mechanisms.

**Fig 1:**
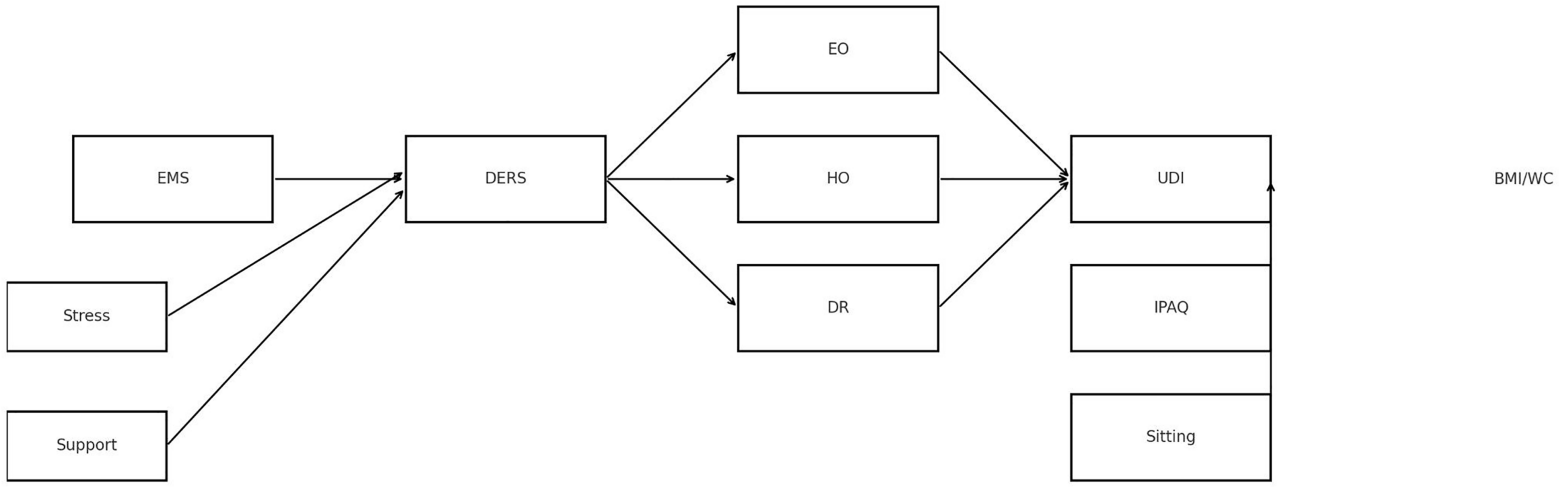
Conceptual model of hypothesized associations among early maladaptive schemas (EMS), emotion regulation difficulties (DERS), contextual factors (perceived stress and social support), eating behavior patterns (emotional overeating [EO], habitual overeating [HO], dietary restraint [DR]), lifestyle indicators (physical activity and sedentary behavior), and adiposity outcomes (body mass index and waist circumference). Arrows represent associations estimated in cross-sectional data rather than causal effects.

Fig 1 depicts the hypothesized associations guiding the present study.

### The present study

The present study aimed to evaluate a comprehensive, integrative model linking EMS, ER difficulties, perceived stress, social support, eating behaviors, diet quality, physical activity, and adiposity in a large adult community sample (N = 1500). Adiposity was operationalized using two correlated outcomes: body mass index (BMI) and waist circumference (WC), with the latter included because of its closer association with central obesity and cardiometabolic risk. Diet quality was assessed using the Unhealthy Diet Index (UDI) derived from the FFQ-6, and physical activity/sedentary behavior using the IPAQ.

The study additionally applied LPA to identify subgroups characterized by distinct configurations of psychological and lifestyle factors. Profiles were compared on adiposity indicators to evaluate both theoretical (EMS, ER, stress, support) and behavioral validation (EO, HO, DR, UDI, activity, sedentary time).

## Hypotheses

- **H1.** Early maladaptive schemas (EMS) will be positively associated with difficulties in emotion regulation (DERS).
- **H2.** Difficulties in emotion regulation (DERS) will be associated with maladaptive eating behaviors, including emotional overeating (EO), habitual overeating (HO), and dietary restraint (DR).
- **H3a.** Maladaptive eating behaviors (EO, HO, and DR) will be associated with adiposity indicators, including body mass index (BMI) and waist circumference (WC).
- **H3b.** Selected maladaptive eating behaviors will mediate the associations between difficulties in emotion regulation (DERS) and adiposity indicators (BMI, WC).
- **H4.** Perceived stress will moderate the association between EMS and DERS, such that higher stress will be associated with stronger EMS–DERS associations.
- **H5.** Perceived social support will moderate the association between EMS and DERS, such that higher social support will attenuate the EMS–DERS association.
- **H6.** Structural associations among EMS, DERS, and maladaptive eating behaviors will differ by gender and age group, with stronger associations expected among women and younger adults.
- **H7.** Higher levels of emotional overeating (EO), habitual overeating (HO), and dietary restraint (DR) will be associated with higher scores on the Unhealthy Diet Index (UDI).
- **H8.** Higher UDI scores will be associated with higher BMI and WC.
- **H9.** Higher levels of physical activity and lower levels of sedentary behavior will be associated with lower UDI, BMI, and WC.

### Latent profile hypotheses

- **H10a.** Latent profile analysis will identify multiple profiles reflecting distinct constellations of psychological, behavioral, and lifestyle characteristics.
- **H10b.** The high-risk profile will be characterized by higher BMI and WC compared to other profiles.
- **H10c.** The high-risk profile will show higher levels of EMS, greater difficulties in emotion regulation, higher perceived stress, and lower perceived social support.
- **H10d.** The high-risk profile will also be characterized by higher levels of emotional overeating, habitual overeating, dietary restraint, poorer diet quality (higher UDI), lower physical activity, and higher sedentary time.
- **H10e.** Latent profile membership will vary by gender and age group.

## Methods

### Participants and procedure

A total of 1,500 adults aged 18–65 years were recruited between January 2018 and February 2025 through advertisements in local universities, healthcare centers (including a specialized obesity clinic), and sports facilities. Recruitment was conducted as part of long-term institutional collaborations to ensure access to diverse populations differing in health and lifestyle. To secure balanced statistical comparisons, participants were stratified by gender, age (younger adults: 18–35 years; older adults: 36–65 years), and body mass index (normal weight vs. overweight/obese). The study employed a non-probability, stratified purposive sampling approach designed to ensure balanced representation across gender, age groups, and BMI categories; accordingly, no statistical weighting was applied, and findings should be interpreted in relation to this stratified community sample rather than as population-representative estimates.

Importantly, no data collection took place during the strict COVID-19 lockdown in 2020, a period that could have substantially affected participants’ emotional functioning and eating behaviors.

Eligibility criteria included being at least 18 years old, able to understand study procedures, and provide informed consent. Individuals with underweight (BMI < 18.5 kg/m²) were excluded due to insufficient representation. Detailed inclusion and exclusion criteria, including psychiatric and medical screening procedures, are provided in S1 File.

All participants completed validated self-report questionnaires in online or paper–pencil format and underwent standardized anthropometric assessments (height, weight, waist circumference) conducted by trained researchers. The study adhered to the Declaration of Helsinki and was approved by the Ethics Committee for Scientific Research at the University of Warmia and Mazury in Olsztyn, Poland (approval no. 1/2017). All participants provided written informed consent prior to participation. This article follows the STROBE guidelines, with the completed checklist provided in S2 File.

### Measures

#### Early Maladaptive Schemas (EMS).

EMS were assessed with the Polish adaptation of the Young Schema Questionnaire – Short Form, Third Edition (YSQ-S3) [1,39]. The 90-item YSQ-S3 measures 18 schemas across five domains on a 6-point Likert scale. Higher scores reflect stronger schema endorsement. Reliability in the present sample was excellent (α = .94). Full CFA loadings for all EMS indicators are provided in S3 File.

#### Emotion regulation difficulties.

Emotion regulation was measured with the Polish version of the Difficulties in Emotion Regulation Scale (DERS; 36 items; α = .93) [7,40].

#### Perceived stress.

Stress was assessed using the Polish version of the Perceived Stress Scale – 10 items (PSS-10; α = .86) [36,41].

#### Perceived social support.

Social support was measured with the Polish version of the Multidimensional Scale of Perceived Social Support (MSPSS; 12 items; α = .92) [38,42].

#### Eating behaviors.

Eating patterns were assessed with the Questionnaire of Eating-Related Behaviors (QERB), measuring emotional overeating (EO), habitual overeating (HO), and dietary restraint (DR) on a 0–10 scale (α = .78–.84) [43].

#### Dietary quality.

FFQ-6 was used to calculate the Unhealthy Diet Index (UDI), reflecting frequency of consuming energy-dense, nutrient-poor foods (α = .80) [23,44].

#### Physical activity and sedentary behavior.

Physical activity and sedentary behavior were measured with the Polish version of the IPAQ-SF. Weekly activity was converted to MET-min/week (IPAQ_MET); sedentary behavior was average daily sitting time (minutes/day). Test–retest reliability is good (r ≈ .80) [25,45].

#### Anthropometric indicators.

BMI was calculated as weight (kg)/height^2^ (m^2^). Waist circumference (WC) was measured at the midpoint between the iliac crest and the lower rib margin and was analyzed separately due to its stronger association with cardiometabolic risk [46].

### Analytic strategy

#### Preliminary analyses.

Preliminary descriptive analyses (normality testing, descriptive statistics, and correlations) were initially conducted in Python and subsequently verified in R to ensure analytical consistency. Descriptive statistics reported in the present article are based on the same cleaned dataset used for all analyses conducted on this cohort, ensuring consistency in reported sample characteristics. For multivariate modeling, selected psychological, behavioral, and contextual variables were represented using composite indicators and standardized scores rather than raw scale totals.

#### Measurement Models (CFA).

Confirmatory factor analyses (CFA) were conducted to validate the latent constructs. EMS was modeled as a higher-order factor indicated by 18 YSQ subscales; DERS as a unidimensional latent factor; and social support as a latent construct indicated by MSPSS items. Model fit was assessed using χ², CFI, TLI, RMSEA, and SRMR. Thresholds of CFI/TLI ≥ .90, RMSEA ≤ .08, and SRMR ≤ .08 were applied as indicators of acceptable fit. Full CFA loadings and residuals for all latent variables are provided in S3 File. Given their well-established unidimensional structure and extensive prior validation, DERS and MSPSS were modeled as single latent constructs without separate CFA reporting; reliability indices are reported in the Measures section.

#### Structural Equation Modeling (SEM).

SEM was applied to examine hypothesized associations linking EMS, DERS, eating behaviors (EO, HO, DR), diet quality (UDI), lifestyle indicators (IPAQ_MET; Sitting), and adiposity outcomes (BMI, WC). Missing data were handled using FIML; outliers were inspected using robust estimation.

Indirect effects were estimated with bias-corrected bootstrapping (5,000 resamples, 95% CIs). Moderation was tested using mean-centered interaction terms (EMS × Stress; EMS × Social Support) predicting DERS. Multi-group invariance across sex and age (configural, metric, scalar) was evaluated, followed by Wald tests of group differences.

Because some fit indices indicated suboptimal fit in the final SEM (CFI = .88; TLI = .86; SRMR = .13), additional model comparisons were performed. More parsimonious alternatives (e.g., models with selected structural paths removed) did not yield meaningful improvements in fit while reducing theoretical coherence; therefore, the full theoretically driven model was retained. All standardized path coefficients, indirect effects, and moderation estimates are reported in S4 File. Because only one higher-order latent factor was specified for EMS, no latent correlations among EMS factors were estimated or reported.

#### Latent Profile Analysis (LPA).

LPA was conducted on z-scores of EMS, DERS, stress, social support, EO, HO, DR, UDI, IPAQ_MET, and Sitting. Solutions with one to six classes were estimated using multiple random starts. Model selection relied on BIC, SABIC, entropy, and the Lo–Mendell–Rubin and bootstrap likelihood-ratio tests. Although three-class solutions showed slightly improved information criteria, the smallest class comprised <5% of participants and lacked theoretical interpretability. The retained two-class solution demonstrated high entropy (≥.80) and clear psychological–behavioral coherence. Fit statistics and class characteristics are reported in S5 File and in the detailed LPA supplements (S7–S9 File).

Class membership was examined using R3STEP, and distal outcomes (BMI, WC) were compared using BCH, with additional psychological and behavioral validators.

### Software

Primary analyses were conducted in R (v4.3.1) using *lavaan* and *semTools* for SEM and *tidyLPA* for LPA. Preliminary descriptive analyses and data wrangling were carried out in Python (v3.11). Reproducible scripts (with fixed random seeds) are provided in S10 File.

## Results

### Sample characteristics

The analytic sample consisted of 1,500 participants (53.0% women, 47.0% men; 55.0% younger adults, 45.0% older adults). Descriptive statistics for continuous study variables are presented in Table 1, and the distribution of participants by sex, age group, residence, and education is reported in Table 2. All measures demonstrated adequate variability without evidence of floor or ceiling effects. No variables showed departures from normality that would preclude SEM estimation.

**Table 1:** Descriptive statistics for continuous variables.

Variable	M	SD	Min	Max
EMS_z	0.01	1.00	–2.85	3.12
DERS_z	0.00	1.00	–2.76	3.21
EO	5.69	1.31	2.10	9.80
HO	5.72	1.33	2.00	9.70
DR	5.66	1.34	2.20	9.60
UDI	0.00	1.00	–2.45	3.01
BMI	25.9	4.8	18.5	35.0
WC (cm)	90.2	14.7	60.0	131.1
Stress (PSS-10)	17.2	6.1	4	38
Social support (MSPSS)	56.8	11.4	24	84

**Note.** EMS_z = standardized Early Maladaptive Schemas; DERS_z = standardized Difficulties in Emotion Regulation; EO = Emotional Overeating; HO = Habitual Overeating; DR = Dietary Restraint; UDI = Unhealthy Diet Index; BMI = Body Mass Index; WC = Waist Circumference; PSS-10 = Perceived Stress Scale; MSPSS = Multidimensional Scale of Perceived Social Support. Higher scores reflect greater symptom severity or higher levels of the construct.

**Table 2:** Sample composition by gender, age group, residence, and education.

Group	n	%
Women	795	53.0
Men	705	47.0
Younger (18–35 y)	825	55.0
Older (36–65 y)	675	45.0
Urban	870	58.0
Rural	630	42.0
Higher education	675	45.0
Secondary education	825	55.0

**Note.** Percentages are based on the total sample (N = 1,500). Values may not sum to 100% due to rounding. Younger adults = 18–35 years; older adults = 36–65 years.

### Measurement models

Confirmatory factor analyses supported the latent structure of EMS. EMS showed adequate higher-order loadings (.59–.67). Fit indices met acceptable thresholds (CFI ≥ .91, RMSEA ≤ .07, SRMR ≤ .07).

DERS and perceived social support were modeled as single latent constructs, consistent with prior validation studies. Full measurement model details are provided in S3 File.

### Structural models (SEM; H1–H9)

The hypothesized SEM demonstrated acceptable but suboptimal fit (χ²(407) = 3303, p < .001; CFI = 0.88; TLI = 0.86; RMSEA = 0.07; SRMR = 0.13). Such indices are common in multi-domain models estimated in large samples, and the structure was retained based on theoretical justification.

EMS were strongly associated with DERS (β = 0.71, p < .001; H1). Higher DERS were associated with emotional overeating and habitual overeating and showed a small negative association with dietary restraint (β ≈ –.09). Given significant gender differences in multi-group models, pooled coefficients should be interpreted with caution.

Direct EMS → EO/HO/DR paths remained significant, indicating partial mediation (H2–H3). The large standardized direct paths from EMS to eating outcomes likely reflect shared variance among self-reported constructs; additional model diagnostics are provided in S3–S4 Files.

Eating behaviors were positively associated with UDI (β = 0.15–0.31, all p < .001; H7). Higher UDI was associated with higher BMI (β = 0.20, p < .001) and WC (β = 0.11, p < .001; H8). Associations with WC were modest.

HO and DR also showed direct relationships with BMI and WC independent of UDI (β = 0.23–0.31).

Physical activity was inversely associated with BMI and WC (β = –0.11 and –0.07), while sedentary time was positively related (β = 0.20 and 0.12; all ps < .001) (Table 3).

**Table 3:** Standardized path coefficients in the structural model.

Path	β	p
EMS → DERS	0.71	<.001
DERS → DR	–0.09	<.001
EMS → EO	0.88	<.001
EMS → HO	0.85	<.001
EMS → DR	0.93	<.001
EO → UDI	0.22	<.001
HO → UDI	0.30	<.001
DR → UDI	0.15	<.001
UDI → BMI	0.20	<.001
UDI → WC	0.11	<.001
HO → BMI	0.26	<.001
HO → WC	0.31	<.001
DR → BMI	0.23	<.001
DR → WC	0.26	<.001
IPAQ_MET → BMI	–0.11	<.001
IPAQ_MET → WC	–0.07	<.001
Sitting → BMI	0.20	<.001
Sitting → WC	0.12	<.001

**Note.** EMS = Early Maladaptive Schemas; DERS = Difficulties in Emotion Regulation; EO = Emotional Overeating; HO = Habitual Overeating; DR = Dietary Restraint; UDI = Unhealthy Diet Index; BMI = Body Mass Index; WC = Waist Circumference; IPAQ_MET = metabolic equivalent minutes per week; Sitting = average daily sedentary time. All coefficients are standardized. All paths shown were estimated simultaneously within the SEM framework using robust maximum likelihood and full-information maximum likelihood for missing data.

Full SEM output including alternative models is reported in S4 File.

### Indirect effects

Indirect effects through DERS and UDI were significant but small for EMS → DR → UDI → BMI/WC (β = –0.002 to –0.003, p < .05).

These effects were statistically detectable but very small in magnitude.

No significant indirect effects emerged for EO or HO.

### Moderation analyses (H4–H5)

The EMS × Stress interaction was nonsignificant (β = 0.02, p =.310; H4 unsupported).

The EMS × Support interaction was significant (β = –0.05, p = .007; H5 supported), indicating weaker EMS → DERS associations among individuals with higher perceived support.

The effect was small and interpreted as a subtle attenuation (Table 4).

**Table 4:** Predictors of DERS with interaction terms.

Predictor	β	p
EMS	0.66	<.001
Stress	0.07	.001
Support	–0.08	<.001
EMS × Stress	0.02	.310
EMS × Support	–0.05	.007

**Note.** Dependent variable = DERS. EMS = Early Maladaptive Schemas; Stress = Perceived Stress (PSS-10); Support = Perceived Social Support (MSPSS). Interaction terms were computed using mean-centered variables. All coefficients are standardized.

A visualization is presented in S1 Fig.

### Multi-group SEM (H6)

Age-group invariance (configural, metric, scalar) was supported; no structural paths differed significantly between younger and older adults.

Gender-group models showed configural invariance but significant structural differences. DERS → EO and DERS → HO paths were stronger among women (Table 5):

**Table 5:** Standardized path coefficients by gender.

Path	Women	Men	p (Wald)
DERS ~ EMS	0.67	0.66	.542
EO ~ DERS	0.63	0.58	.033
HO ~ DERS	0.65	0.58	<.001
DR ~ DERS	–0.09	–0.08	.418

**Note.** Standardized coefficients from multi-group SEM. Wald tests compare structural path equality across gender groups. EMS = Early Maladaptive Schemas; DERS = Difficulties in Emotion Regulation; EO = Emotional Overeating; HO = Habitual Overeating; DR = Dietary Restraint.

### Latent profile analysis (H10a–H10e)

#### Model selection.

LPA models with 1–5 classes were estimated. The two-class solution demonstrated the best overall fit, the lowest BIC, high entropy (0.94), and no class comprising fewer than 5% of participants. This solution was selected based on model fit, class size, and interpretability. Fit indices and model comparisons are provided in S5 File.

#### Class description (H10a).

- Class 1 (High-risk): higher EMS, higher DERS, higher stress, lower social support; higher EO, HO, DR, and UDI; lower activity and higher sedentary time.
- Class 2 (Low-risk/Buffered): opposite profile.

Detailed descriptive comparisons provided in S7–S10 File.

### Adiposity differences (H10b)

High-risk individuals showed higher BMI (29.8 vs. 21.8) and WC (99.8 vs. 80.0 cm; p < .001).

Outputs in S7 File.

### Psychological & behavioral validation (H10c–H10d)

High-risk participants had higher EMS, DERS, stress, EO, HO, DR, UDI, lower activity, and higher sedentary time (all p < .001).

Full comparisons in S8–S10 File.

### Demographic covariates (H10e)

Female gender (OR = 1.42) and younger age (OR = 1.36) predicted membership in the High-risk class.

## Summary of hypotheses testing

H1 — supported
H2–H3 — partially supported
H4 — not supported
H5 — supported
H6 — partially supported
H7–H9 — supported
H10a–H10d — supported
H10e — supported (exploratory demographic covariates)

## Discussion

The present study tested a comprehensive SEM linking EMS, ER difficulties, stress, social support, maladaptive eating behaviors, diet quality, physical activity, and adiposity outcomes in a large adult sample. By simultaneously incorporating cognitive–emotional, contextual, and lifestyle variables, the study provides an integrative framework for understanding the multifactorial pathways associated with obesity. Overall, the findings were largely consistent with the hypothesized associations and highlighted the relevance of psychological vulnerabilities and lifestyle behaviors as joint correlates of dietary and adiposity outcomes. Although model fit did not meet optimal thresholds (CFI/TLI < .90; SRMR > .08), this is not uncommon for complex, multi-domain SEMs in large samples. Alternative models were examined to ensure pathway stability, and the final integrated model was retained to preserve theoretical coherence despite suboptimal fit indices. The presence of consistent but mostly small effects, alongside some moderate direct associations, suggests that the retained model captures meaningful relationships, while also indicating that additional variables (e.g., sleep, socioeconomic status, environmental factors) are likely to account for residual variance beyond the constructs included here. These considerations guided the interpretation of the results presented below.

### Cognitive–emotional mechanisms

As hypothesized (H1), EMS were strongly associated with ER difficulties, reinforcing schema theory that positions EMS as enduring cognitive–emotional patterns compromising affect regulation [1,2]. This indicates that maladaptive schemas are not only distal vulnerabilities but remain linked to emotion–regulation capacities in adulthood. Consistent with H2, ER difficulties partly mediated the association between EMS and eating behavior, but this indirect effect was selective and limited primarily to dietary restraint; no indirect effects emerged for emotional or habitual overeating. Importantly, EMS also showed direct associations with emotional overeating, habitual overeating, and dietary restraint, suggesting that maladaptive schemas may serve as cognitive templates for eating dysregulation. These results converge with prior evidence that ER difficulties partly explain links between schemas and eating pathology [9,47], while also indicating that schema–eating associations are predominantly direct rather than broadly mediated by ER deficits.

Indirect pathways from EMS to adiposity via dietary restraint and UDI were statistically significant but very small and limited to the pathway involving dietary restraint and diet quality**,** indicating that emotion-related processes may be more proximal to eating patterns than to weight status itself. Longitudinal studies similarly show that individuals with elevated ER difficulties are more prone to emotional eating and poorer diet quality, which in turn predict weight gain over time [12–14]. The present study adds cross-sectional evidence from a large adult sample showing similar patterns across both adiposity indicators (BMI and WC), while underscoring that effect sizes for indirect links to adiposity should be interpreted cautiously. Importantly, the selective and modest mediating role of emotion regulation difficulties observed in the present study is theoretically consistent with the inclusion of multiple behavioral and lifestyle pathways within the same integrative model. Emotion regulation represents a relatively proximal mechanism shaping eating behavior, whereas adiposity indicators such as body mass index and waist circumference reflect cumulative, long-term outcomes influenced by a broad constellation of behavioral and contextual factors. When diet quality, physical activity, and sedentary behavior are modeled simultaneously, the contribution of emotion regulation to distal adiposity outcomes is therefore expected to attenuate rather than dominate the overall model. In this context, early maladaptive schemas may influence eating behavior not only through emotion regulation difficulties, but also via more direct cognitive and behavioral pathways, including self-control, impulsivity, normative beliefs about eating, and coping strategies.

### Lifestyle and diet quality

An important contribution of the present study is the simultaneous integration of cognitive–emotional vulnerabilities with lifestyle behaviors and contextual factors within a single analytical framework. As hypothesized (H7–H9), emotional overeating, habitual overeating, and dietary restraint were linked to higher scores on the UDI, which in turn was associated with elevated BMI and WC. These findings suggest that self-reported maladaptive eating tendencies correspond with actual dietary patterns, strengthening the ecological validity of psychological models of eating. Physical activity (IPAQ_MET) and sedentary time (Sitting) added further explanatory value. Higher physical activity was associated with lower adiposity, while greater sedentary behavior was related to higher BMI and WC. These associations align with a large body of evidence emphasizing the dual importance of diet quality and physical activity for weight outcomes [24,25,48]. Importantly, SEM results indicated that lifestyle behaviors were not merely confounding factors but operated as parallel pathways complementing psychological mechanisms. This multidimensional approach highlights that obesity risk is shaped by the intersection of schemas, emotion regulation, stress, social context, and concrete health behaviors.

### Contextual moderators: Stress and social support

Moderation analyses yielded partial support for the hypothesized contextual effects. Contrary to H4, perceived stress did not significantly moderate the EMS–ER association. This pattern suggests that, although stress is a robust correlate of eating dysregulation and weight gain in prior work [16,17], its influence may operate more directly on eating behaviors rather than by amplifying the EMS–ER link; similar null moderation results have been reported elsewhere for schema–emotion and emotion–eating pathways [49,50]. In contrast, social support significantly moderated the EMS–ER pathway (H5): higher support attenuated the EMS–ER link. Individuals with lower perceived support exhibited stronger associations between EMS and ER difficulties, consistent with evidence that social ties buffer psychological vulnerability and improve health outcomes [51–53], as well as findings that supportive relationships reduce reliance on maladaptive coping such as emotional eating [19]. However, the size of this interaction effect was small, indicating a subtle rather than a pronounced buffering influence. Together, these results highlight social support as a critical resilience factor capable of offsetting risks associated with early maladaptive schemas and underscore the potential of interpersonal and community resources to mitigate psychological vulnerabilities in the context of obesity.

### Gender and age differences

Consistent with H6, multi-group SEM analyses showed that the associations of ER difficulties with both emotional overeating and habitual overeating were significantly stronger among women than men. This pattern aligns with longstanding evidence that women are more prone to emotion-driven eating [50,54] and supports previous findings from the same cohort showing higher schema endorsement and emotional eating in women compared to men [5]. These results underline the importance of considering gender-specific vulnerabilities when designing preventive and therapeutic interventions, as schema- and ER-focused strategies may be particularly relevant for women. No significant age-related differences were observed in structural paths. Taken together with prior evidence showing higher mean levels of schemas and emotional eating in younger adults [3,4], this suggests that age effects may be more evident in overall vulnerability levels rather than in the structural interrelations among schemas, emotion regulation, and eating behaviors.

### Latent profile analysis

The latent profile analysis identified two distinct classes representing lower- and higher-risk constellations of psychological, behavioral, and lifestyle characteristics. The emergence of a two-class solution is consistent with methodological evidence showing that the number and structure of latent profiles are sensitive to indicator selection and model specification. In the present analysis, lifestyle and contextual indicators—including diet quality, physical activity, sedentary behavior, perceived stress, and social support—were incorporated alongside cognitive–emotional variables, yielding an empirically optimal solution within the expected range of two to three profiles.

When these additional indicators were modeled simultaneously with early maladaptive schemas and emotion regulation difficulties, participant profiles polarized into a robust high- versus low-risk configuration, with intermediate patterns no longer forming a stable class. Instead, individuals clustered into two clearly differentiated constellations reflecting protective versus maladaptive patterns across cognitive, emotional, behavioral, and lifestyle domains. This pattern suggests that the inclusion of lifestyle and contextual variables amplifies risk differentiation, revealing sharper contrasts between profiles than models based solely on psychological indicators.

### Theoretical contribution and interpretive scope

From a theoretical perspective, the present findings advance integrative models of eating behavior and obesity by clarifying the selective and context-dependent pathways linking early maladaptive schemas, emotion regulation difficulties, contextual resources, and lifestyle behaviors. Rather than supporting a universal mediational role of emotion regulation, the results indicate that cognitive–emotional vulnerabilities operate primarily through robust direct associations with eating behaviors, with only limited and pathway-specific indirect effects.

Importantly, this pattern underscores the distinction between proximal psychological mechanisms shaping eating behavior and more distal indicators of adiposity, such as body mass index and waist circumference, which reflect the cumulative influence of multiple behavioral, metabolic, and contextual processes over time. When lifestyle behaviors—including diet quality, physical activity, and sedentary behavior—are considered alongside cognitive–emotional and social factors, the explanatory role of emotion regulation difficulties is expected to attenuate rather than dominate the overall model.

The present study therefore contributes to theory by delineating which mechanisms remain salient when psychological vulnerabilities, contextual moderators, and concrete lifestyle behaviors are examined simultaneously within a single integrative framework. Given the non-random sampling design, these findings should be interpreted as theory-informing rather than population-generalizable. Nevertheless, the results offer a coherent conceptual basis for understanding how cognitive–emotional, social, and behavioral factors jointly shape eating patterns and adiposity, while avoiding overgeneralization beyond the empirical scope of the sample.

### Limitations

Despite its strengths, several limitations should be acknowledged. First, the cross-sectional design precludes causal inferences. All pathways were modeled and interpreted as associations rather than causal effects. Longitudinal and experimental studies are needed to determine whether EMS and ER difficulties prospectively relate to changes in eating behavior, diet quality, and adiposity. Second, most variables were assessed via self-report, which may be subject to recall and social desirability biases, particularly for dietary intake and physical activity. However, anthropometric outcomes (BMI and WC) were measured directly by trained researchers, reducing measurement error for these indices. Including objective measures such as accelerometry or 24-hour dietary recalls would enhance validity. Third, contextual moderators were limited to stress and social support; other important factors such as socioeconomic status, sleep, or cultural norms were not included. Fourth, while the sample was large and balanced by age and gender, it was drawn from a single national context, which may limit generalizability. Additionally, gender was operationalized using a binary classification (women/men), which does not capture gender diversity and may limit the applicability of the findings to non-binary and gender-diverse populations.

Fifth, although model fit indices did not reach conventional thresholds (CFI/TLI < .90; SRMR = .13), such deviations are common in large-sample, multi-domain SEMs that incorporate multiple latent constructs and interaction terms. Prior methodological work has emphasized that fit indices should not be interpreted rigidly and that theoretical coherence and interpretability are equally critical in evaluating SEM solutions [55,56]. In this context, the retained framework provides a substantively meaningful and statistically stable representation of the hypothesized pathways, even if statistical indices are not optimal. Finally, although SEM enabled the testing of mediation, moderation, and group differences, the framework required stepwise analyses rather than a fully integrated model, which may have limited the ability to test all higher-order interactions in a single equation system.

## Conclusion

This study demonstrates that early maladaptive schemas are associated with maladaptive eating patterns and adiposity primarily through robust direct relationships, with only small and selective indirect pathways involving emotion regulation difficulties, dietary restraint, and diet quality. Lifestyle behaviors further accounted for variance in adiposity outcomes, while social support emerged as a modest but consistent protective moderator of the association between schemas and emotion regulation difficulties. Gender-specific patterns were also observed, with women exhibiting stronger associations between emotion regulation difficulties and maladaptive eating behaviors.

Taken together, these findings underscore the importance of integrative approaches that consider cognitive–emotional vulnerabilities, contextual resources, and concrete lifestyle behaviors simultaneously when examining pathways to obesity. Rather than implicating a single dominant psychological mechanism, the results highlight the cumulative and interacting contributions of multiple factors, suggesting that effective prevention and intervention efforts should be multidimensional and context-sensitive.

### Implications for practice and policy

These implications should be interpreted as theory-informed considerations rather than direct clinical or policy recommendations. The findings carry several implications:

- **Theory:** Results reinforce schema theory in the domain of eating behavior and obesity, positioning early maladaptive schemas as transdiagnostic vulnerabilities associated with eating-related and adiposity outcomes.
- **Clinical practice:** Interventions may benefit from integrating schema-informed strategies (e.g., addressing maladaptive beliefs, enhancing adaptive coping) with emotion regulation skills training and lifestyle modification. Strengthening social support may further enhance intervention effects.
- **Prevention:** School- and workplace-based health programs could incorporate schema-informed psychoeducation, mindfulness-based approaches, and group-based emotion regulation skills training to foster resilience against maladaptive eating patterns.
- **Policy:** Public health initiatives may benefit from addressing psychological vulnerabilities alongside lifestyle behaviors, embedding schema- and emotion-regulation-informed components into obesity prevention strategies. Enhancing social support networks within communities may represent a scalable protective resource.
- **Demographic tailoring:** Preventive and intervention efforts should be gender-sensitive, with particular attention to women, who demonstrated stronger associations between emotion regulation difficulties and maladaptive eating behaviors.

These findings are consistent with contemporary biopsychosocial perspectives emphasizing the dynamic interplay between cognitive–emotional, social, and behavioral processes in health and disease [57–59]. Overall, the study advances an integrative biopsychosocial model of obesity, demonstrating how maladaptive schemas and emotion regulation difficulties intersect with stress, social support, diet, and physical activity in shaping adiposity-related outcomes. This multidimensional perspective may inform more targeted and context-sensitive approaches to prevention and intervention.

## Supporting information

S1 File Inclusion and exclusion criteria. Detailed eligibility criteria, including psychiatric and medical exclusion procedures, and description of recruitment stratification (gender, age, BMI groups).
(DOCX)

S2 File STROBE checklist. Completed checklist documenting adherence to the Strengthening the Reporting of Observational Studies in Epidemiology (STROBE) guidelines.
(DOCX)

S3 File Measurement model details (CFA for EMS). Confirmatory factor analysis results for Early Maladaptive Schemas (EMS), including standardized factor loadings with standard errors and test statistics.
(DOCX)

S4 File Structural equation modeling (SEM) details. Complete SEM output including standardized and unstandardized path coefficients, indirect effects, and moderation estimates.
(DOCX)

S5 File Latent profile model fit indices. Fit statistics for 1–5-class latent profile models (BIC, SABIC, entropy, LMR, BLRT), with comparison tables identifying the optimal two-class solution.
(DOCX)

S6 File Latent profile descriptions. Standardized means (z-scores) of psychological, behavioral, and lifestyle variables across the identified latent profiles (High-risk and Low-risk classes).
(DOCX)

S7 File Demographic predictors of latent profile membership. Logistic regression analyses examining associations of sex and age with membership in the High-risk versus Low-risk latent profile.
(DOCX)

S8 File Psychological validators. Between-class comparisons for EMS, DERS, perceived stress, and social support with full test statistics and effect sizes.
(DOCX)

S9 File Behavioral validators. Between-class comparisons for eating behaviors (EO, HO, DR), Unhealthy Diet Index (UDI), physical activity (IPAQ_MET), and sedentary behavior (Sitting).
(DOCX)

S10 File Statistical software and example analysis code. Example R and Python code illustrating data preparation, CFA, SEM, multi-group analyses, and latent profile analysis procedures used in the study.
(DOCX)

S1 Fig Interaction between early maladaptive schemas (EMS) and perceived social support in relation to difficulties in emotion regulation (DERS). Values are standardized (z-scores). Lines represent estimated simple slopes at −1 SD (low social support), mean, and +1 SD (high social support).
(PDF)

## Abbreviations

BMI: body mass index
WC: waist circumference
EMS: early maladaptive schemas
ER: emotion regulation
DERS: difficulties in emotion regulation scale
YSQ: young schema questionnaire
MSPSS: multidimensional scale of perceived social support
PSS: perceived stress scale
QERB: questionnaire of eating-related behaviors
EO: emotional overeating
HO: habitual overeating
DR: dietary restraint
UDI: unhealthy diet index
IPAQ: international physical activity questionnaire
MET: metabolic equivalent of task
SEM: structural equation modeling
CFA: confirmatory factor analysis
LPA: latent profile analysis
FIML: full information maximum likelihood
BIC: Bayesian information criterion
SABIC: sample-size adjusted Bayesian information criterion
RMSEA: root mean square error of approximation
SRMR: standardized root mean square residual
CFI: comparative fit index
TLI: Tucker–Lewis index
