# Supplementary material for: Psychological and lifestyle correlates of eating behavior and adiposity: Structural and latent profile modeling
Source: PLoS One. 2026 Feb 20;21(2):e0343336. doi: 10.1371/journal.pone.0343336 (PMC12922993; doi:10.1371/journal.pone.0343336)
Supplement: S1 File — Detailed eligibility criteria, including psychiatric and medical exclusion procedures, and description of recruitment stratification (gender, age, BMI groups). (DOCX) [file pone.0343336.s001.docx]

**Supplementary File 1. Inclusion and Exclusion Criteria**

**Inclusion criteria**

- Age 18–65 years
- BMI in the normal weight (18.5–24.9 kg/m²) or overweight/obese range (≥25 kg/m²)
- Ability to understand study procedures and provide informed consent
- Recruitment from community (universities, sports facilities) and medical contexts (healthcare centers, obesity treatment clinic)

**Exclusion criteria**

- BMI <18.5 kg/m² (underweight, n = 9, excluded)
- Pregnancy or lactation
- Legal incapacity or inability to provide informed consent
- Severe psychiatric disorders (schizophrenia, bipolar disorder, history of psychiatric hospitalization for severe mental disorders)
- Ongoing psychiatric treatment for severe conditions
- Intellectual disabilities interfering with questionnaire completion
- Substance dependence (alcohol or drugs)
- Severe or uncontrolled chronic medical conditions significantly affecting weight regulation (e.g., type 1 diabetes, untreated or uncontrolled thyroid disease, rare metabolic disorders of known endocrine or genetic origin). Common, medically treated conditions such as type 2 diabetes or controlled thyroid disorders were not exclusionary.

**Screening procedure**
Participants completed a brief, structured medical history questionnaire developed for the purposes of the study, based on routine outpatient psychiatric screening questions commonly used in clinical practice in Poland. The questionnaire included items on:
• prior psychiatric diagnoses
• psychiatric hospitalizations
• current psychiatric treatment
• substance use history

The screening procedure was used solely to identify and exclude individuals reporting severe psychiatric disorders or recent psychiatric hospitalization and was not intended as a diagnostic or psychometric assessment.
