# Supplementary material for: Psychological and lifestyle correlates of eating behavior and adiposity: Structural and latent profile modeling
Source: PLoS One. 2026 Feb 20;21(2):e0343336. doi: 10.1371/journal.pone.0343336 (PMC12922993; doi:10.1371/journal.pone.0343336)
Supplement: S2 File — Completed checklist documenting adherence to the Strengthening the Reporting of Observational Studies in Epidemiology (STROBE) guidelines. (DOCX) [file pone.0343336.s002.docx]

**Supplementary File 2. STROBE Statement—Checklist of items that should be included in reports of cross-sectional studies**

| **Item** | **Recommendation** | **Section of manuscript** |
| --- | --- | --- |
| **Title and abstract** | **1a.** Indicate the study’s design with a commonly used term in the title or the abstract. | Title page, Abstract |
|  | **1b.** Provide in the abstract an informative and balanced summary of what was done and what was found. | Abstract |
| **Introduction** | **2.** Explain the scientific background and rationale for the investigation being reported. | Introduction |
|  | **3.** State specific objectives, including any prespecified hypotheses. | Introduction (The Present Study, Hypotheses) |
| **Methods** | **4.** Present key elements of study design early in the paper. | Methods |
|  | **5.** Describe the setting, locations, and relevant dates, including periods of recruitment, exposure, follow-up, and data collection. | Participants and Procedure |
|  | **6a.** Give the eligibility criteria, and the sources and methods of selection of participants. | Participants and Procedure; S1 File |
|  | **7.** Clearly define all outcomes, exposures, predictors, potential confounders, and effect modifiers. Give diagnostic criteria, if applicable. | Measures |
|  | **8.** For each variable of interest, give sources of data and details of methods of assessment (measurement). Describe comparability of assessment methods if there is more than one group. | Measures |
|  | **9.** Describe any efforts to address potential sources of bias. | Participants and Procedure; Analytic Strategy; Discussion (Limitations) |
|  | **10.** Explain how the study size was arrived at. | Participants and Procedure (community sample; sample size determined by recruitment feasibility) |
|  | **11.** Explain how quantitative variables were handled in the analyses. If applicable, describe which groupings were chosen and why. | Analytic Strategy |
|  | **12a.** Describe all statistical methods, including those used to control for confounding. | Analytic Strategy |
|  | **12b.** Describe any methods used to examine subgroups and interactions. | Analytic Strategy (moderation, multi-group SEM, latent profile analysis) |
|  | **12c.** Explain how missing data were addressed. | Analytic Strategy (FIML approach in SEM; winsorization/robust methods); S10 File |
|  | **12d.** If applicable, explain how matching of cases and controls was addressed. | Not applicable |
|  | **12e.** Describe any sensitivity analyses. | Not applicable |
| **Results** | **13a.** Report numbers of individuals at each stage of study—e.g., numbers potentially eligible, examined for eligibility, confirmed eligible, included in the study, completing follow-up, and analysed. | Participants and Procedure; S1 File |
|  | **13b.** Give reasons for non-participation at each stage. | Participants and Procedure; S1 File |
|  | **13c.** Consider use of a flow diagram. | Not applicable (no flow diagram prepared) |
|  | **14a.** Give characteristics of study participants (e.g., demographic, clinical, social) and information on exposures and potential confounders. | Results (Tables 1a and 1b) |
|  | **14b.** Indicate number of participants with missing data for each variable of interest. | Results (Table 1a) |
|  | **15.** Report numbers of outcome events or summary measures. | Results (Tables 2–4, text) |
|  | **16a.** Give unadjusted estimates and, if applicable, confounder-adjusted estimates and their precision (e.g., 95% confidence interval). Make clear which confounders were adjusted for and why they were included. | Results (Tables 2–4; multivariable SEM models with standardized estimates and confidence intervals) |
|  | **16b.** Report category boundaries when continuous variables were categorized. | Participants and Procedure (BMI categories, age group definitions) |
|  | **16c.** If relevant, consider translating estimates of relative risk into absolute risk. | Not applicable |
|  | **17.** Report other analyses done—e.g., analyses of subgroups and interactions, and sensitivity analyses. | Results (moderation analyses, multi-group SEM, latent profile analysis) |
| **Discussion** | **18.** Summarise key results with reference to study objectives. | Discussion |
|  | **19.** Discuss limitations of the study, taking into account sources of potential bias or imprecision. Discuss both direction and magnitude of any potential bias. | Discussion (Limitations) |
|  | **20.** Give a cautious overall interpretation of results considering objectives, limitations, multiplicity of analyses, results from similar studies, and other relevant evidence. | Discussion |
|  | **21.** Discuss the generalisability (external validity) of the study results. | Discussion (Implications and generalisability) |
| **Other information** | **22.** Give the source of funding and the role of the funders for the present study. | Provided during submission (Funding information entered in the journal’s submission system). |
