# Supplementary material for: Psychological and lifestyle correlates of eating behavior and adiposity: Structural and latent profile modeling
Source: PLoS One. 2026 Feb 20;21(2):e0343336. doi: 10.1371/journal.pone.0343336 (PMC12922993; doi:10.1371/journal.pone.0343336)
Supplement: S3 File — Confirmatory factor analysis results for Early Maladaptive Schemas (EMS), including standardized factor loadings with standard errors and test statistics. (DOCX) [file pone.0343336.s003.docx]

**S3 Table. CFA Standardized Loadings for Early Maladaptive Schemas (EMS)**

| **Item** | **Std. Loading** | **SE** | **z** | **p** |
| --- | --- | --- | --- | --- |
| Emotional Deprivation | 0.653 | — | — | — |
| Abandonment | 0.595 | 0.042 | 21.19 | <.001 |
| Mistrust/Abuse | 0.627 | 0.043 | 22.14 | <.001 |
| Social Isolation | 0.670 | 0.043 | 23.50 | <.001 |
| Defectiveness/Shame | 0.650 | 0.044 | 22.90 | <.001 |
| Failure | 0.645 | 0.042 | 22.72 | <.001 |
| Dependence/Incompetence | 0.625 | 0.041 | 22.07 | <.001 |
| Vulnerability to Harm | 0.631 | 0.042 | 22.23 | <.001 |
| Enmeshment | 0.591 | 0.041 | 21.00 | <.001 |
| Subjugation | 0.590 | 0.042 | 20.97 | <.001 |
| Self-Sacrifice | 0.615 | 0.041 | 21.80 | <.001 |
| Approval Seeking | 0.655 | 0.043 | 23.02 | <.001 |
| Negativity/Pessimism | 0.667 | 0.043 | 23.37 | <.001 |
| Emotional Inhibition | 0.633 | 0.042 | 22.29 | <.001 |
| Unrelenting Standards | 0.669 | 0.042 | 23.40 | <.001 |
| Entitlement | 0.666 | 0.044 | 23.38 | <.001 |
| Insufficient Self-Control | 0.643 | 0.042 | 22.70 | <.001 |
| Punitiveness | −0.037 | 0.038 | −1.38 | .168 |

**Note:** Table S2 reports standardized CFA loadings from the measurement model for the EMS factor. All items load significantly except *Punitiveness*, which shows a near-zero, nonsignificant loading, consistent with previous evidence that this item behaves inconsistently across populations.
