# Supplementary material for: Psychological and lifestyle correlates of eating behavior and adiposity: Structural and latent profile modeling
Source: PLoS One. 2026 Feb 20;21(2):e0343336. doi: 10.1371/journal.pone.0343336 (PMC12922993; doi:10.1371/journal.pone.0343336)
Supplement: S4 File — Complete SEM output including standardized and unstandardized path coefficients, indirect effects, and moderation estimates. (DOCX) [file pone.0343336.s004.docx]

**Table S4. SEM Path Coefficients for the Full Structural Model**

**DERS outcomes**

| **Predictor → Outcome** | **Est** | **SE** | **z** | **p** |
| --- | --- | --- | --- | --- |
| EMS → DERS | **1.373** | 0.068 | 20.24 | <.001 |
| Stress → DERS | 0.033 | 0.025 | 1.33 | .184 |
| Support → DERS | −0.030 | 0.020 | −1.47 | .141 |
| EMS×Stress → DERS | 0.018 | 0.018 | 0.98 | .328 |
| EMS×Support → DERS | **0.063** | 0.022 | 2.92 | .004 |

**Eating behaviors (QERB)**

**Emotional Overeating (EO)**

| **Predictor → EO** | **Est** | **SE** | **z** | **p** |
| --- | --- | --- | --- | --- |
| DERS → EO | −0.013 | 0.022 | −0.59 | .554 |
| EMS → EO | **1.748** | 0.070 | 25.04 | <.001 |

**Habitual Overeating (HO)**

| **Predictor → HO** | **Est** | **SE** | **z** | **p** |
| --- | --- | --- | --- | --- |
| DERS → HO | 0.019 | 0.023 | 0.85 | .394 |
| EMS → HO | **1.691** | 0.069 | 24.65 | <.001 |

**Restraint (DR)**

| **Predictor → DR** | **Est** | **SE** | **z** | **p** |
| --- | --- | --- | --- | --- |
| DERS → DR | **−0.092** | 0.023 | −4.03 | <.001 |
| EMS → DR | **1.856** | 0.073 | 25.59 | <.001 |

**Unhealthy Diet Index (UDI)**

| **Predictor → UDI** | **Est** | **SE** | **z** | **p** |
| --- | --- | --- | --- | --- |
| DERS → UDI | −0.006 | 0.026 | −0.24 | .811 |
| EO → UDI | **0.219** | 0.038 | 5.72 | <.001 |
| HO → UDI | **0.303** | 0.039 | 7.70 | <.001 |
| DR → UDI | **0.148** | 0.037 | 4.00 | <.001 |
| MET → UDI | −0.058 | 0.020 | −2.90 | .004 |
| Sitting → UDI | **0.055** | 0.020 | 2.68 | .007 |

**BMI**

| **Predictor → BMI** | **Est** | **SE** | **z** | **p** |
| --- | --- | --- | --- | --- |
| UDI → BMI | **0.202** | 0.020 | 9.88 | <.001 |
| EO → BMI | **0.076** | 0.030 | 2.53 | .011 |
| HO → BMI | **0.259** | 0.031 | 8.34 | <.001 |
| DR → BMI | **0.231** | 0.029 | 7.83 | <.001 |
| MET → BMI | **−0.106** | 0.016 | −6.72 | <.001 |
| Sitting → BMI | **0.201** | 0.016 | 12.40 | <.001 |

**Waist Circumference (WC)**

| **Predictor → WC** | **Est** | **SE** | **z** | **p** |
| --- | --- | --- | --- | --- |
| UDI → WC | **0.113** | 0.024 | 4.72 | <.001 |
| EO → WC | 0.035 | 0.035 | 0.99 | .324 |
| HO → WC | **0.313** | 0.036 | 8.61 | <.001 |
| DR → WC | **0.256** | 0.035 | 7.39 | <.001 |
| MET → WC | **−0.066** | 0.019 | −3.55 | <.001 |
| Sitting → WC | **0.116** | 0.019 | 6.08 | <.001 |

**Indirect effects**

| **Effect** | **Estimate** | **p** |
| --- | --- | --- |
| EMS → DR → BMI | **−0.003** | .006 |
| EMS → DR → WC | **−0.002** | .015 |
| All other indirect paths | ns | — |

**Note:** Table S4 summarizes all regression paths from the SEM model, including predictors of DERS, eating behaviors, UDI, BMI, and waist circumference. Standardized results follow the pattern reported by the lavaan Std.all column; estimates marked in bold are statistically significant at *p* < .05. Non-significant direct paths from difficulties in emotion regulation to emotional and habitual overeating reflect shared variance with early maladaptive schemas in the full model.
