# Supplementary material for: Psychological and lifestyle correlates of eating behavior and adiposity: Structural and latent profile modeling
Source: PLoS One. 2026 Feb 20;21(2):e0343336. doi: 10.1371/journal.pone.0343336 (PMC12922993; doi:10.1371/journal.pone.0343336)
Supplement: S5 File — Fit statistics for 1–5-class latent profile models (BIC, SABIC, entropy, LMR, BLRT), with comparison tables identifying the optimal two-class solution. (DOCX) [file pone.0343336.s005.docx]

**Supplementary File 5. Latent Profile Analysis (LPA) – Fit Indices**

**Table S5 Model fit indices for 1–5 class solutions**

| **Classes** | **AIC** | **BIC** | **SABIC** | **Entropy** | **LMR p** | **BLRT p** | **Smallest class (%)** |
| --- | --- | --- | --- | --- | --- | --- | --- |
| 1 | 20543.6 | 20688.5 | 20591.4 | – | – | – | 100.0 |
| 2 | 18212.3 | 18398.4 | 18273.9 | 0.94 | <.001 | <.001 | 48.5 |
| 3 | 18201.1 | 18428.4 | 18276.6 | 0.87 | .081 | .094 | 12.0 |
| 4 | 18195.7 | 18464.1 | 18285.0 | 0.79 | .122 | .133 | 6.4 |
| 5 | 18192.2 | 18501.9 | 18295.4 | 0.74 | .147 | .159 | 3.1 |

**Note.** AIC = Akaike Information Criterion; BIC = Bayesian Information Criterion; SABIC = Sample-size Adjusted BIC; LMR = Lo–Mendell–Rubin likelihood ratio test; BLRT = Bootstrap likelihood ratio test.
The 2-class model showed the best balance of fit, entropy, and class size, with no class below 5%.
