# Supplementary material for: Psychological and lifestyle correlates of eating behavior and adiposity: Structural and latent profile modeling
Source: PLoS One. 2026 Feb 20;21(2):e0343336. doi: 10.1371/journal.pone.0343336 (PMC12922993; doi:10.1371/journal.pone.0343336)
Supplement: S6 File — Standardized means (z-scores) of psychological, behavioral, and lifestyle variables across the identified latent profiles (High-risk and Low-risk classes). (DOCX) [file pone.0343336.s006.docx]

**Supplementary File 6. Latent Profile Analysis – Profile Descriptions**

**Table S6. Standardized means of study variables across latent classes**

| **Variable** | **Class 1 (High-risk)** | **Class 2 (Low-risk)** |
| --- | --- | --- |
| EMS | +0.92 | –0.88 |
| DERS | +0.85 | –0.81 |
| Stress (PSS-10) | +0.77 | –0.74 |
| Social Support | –0.69 | +0.71 |
| EO | +0.83 | –0.79 |
| HO | +0.88 | –0.84 |
| DR | +0.64 | –0.61 |
| UDI | +0.91 | –0.86 |
| IPAQ_MET | –0.73 | +0.69 |
| Sitting | +0.81 | –0.76 |

**Note.** Values are standardized (z-scores). Class 1 = *High-risk profile* (psychological vulnerability & unhealthy lifestyle); Class 2 = *Low-risk/Buffered profile* (psychologically buffered & active lifestyle).
