# Supplementary material for: Psychological and lifestyle correlates of eating behavior and adiposity: Structural and latent profile modeling
Source: PLoS One. 2026 Feb 20;21(2):e0343336. doi: 10.1371/journal.pone.0343336 (PMC12922993; doi:10.1371/journal.pone.0343336)
Supplement: S7 File — Logistic regression analyses examining associations of sex and age with membership in the High-risk versus Low-risk latent profile. (DOCX) [file pone.0343336.s007.docx]

**Supplementary File 7. Logistic Regression – Demographic Predictors of Class Membership**

**Table S7. Logistic regression predicting membership in the High-risk profile (vs. Low-risk)**

| **Predictor** | **OR** | **95% CI** | **p** |
| --- | --- | --- | --- |
| Female (vs. male) | 1.42 | 1.18 – 1.71 | <.001 |
| Younger (vs. older) | 1.36 | 1.12 – 1.65 | .002 |

**Note.** OR = Odds Ratio; CI = Confidence Interval. Being female and younger significantly increased the odds of belonging to the High-risk profile.
