# Supplementary material for: Psychological and lifestyle correlates of eating behavior and adiposity: Structural and latent profile modeling
Source: PLoS One. 2026 Feb 20;21(2):e0343336. doi: 10.1371/journal.pone.0343336 (PMC12922993; doi:10.1371/journal.pone.0343336)
Supplement: S8 File — Between-class comparisons for EMS, DERS, perceived stress, and social support with full test statistics and effect sizes. (DOCX) [file pone.0343336.s008.docx]

**S8 File. Psychological validators**

**Between-class comparisons for EMS, DERS, perceived stress, and social support.**

**Table S8. Psychological validators across latent profiles (Low-risk vs High-risk)**

| **Variable** | **Low-risk M (SD)** | **High-risk M (SD)** | **t (Welch)** | **p** | **Hedges g** |
| --- | --- | --- | --- | --- | --- |
| EMS (overall) | 3.63 (0.31) | 4.32 (0.33) | 41.60 | <.001 | 2.15 |
| DERS (total) | 104.62 (10.90) | 118.26 (11.49) | 23.60 | <.001 | 1.22 |
| Perceived stress (PSS-10) | 20.03 (3.73) | 23.88 (3.85) | 19.68 | <.001 | 1.02 |
| Perceived social support (MPSS) | 66.67 (7.83) | 58.26 (7.42) | -21.31 | <.001 | -1.10 |

**Note.** Low-risk profile: LPA_Class = 2 (n = 727). High-risk profile: LPA_Class = 1 (n = 773). Group differences were tested using **Welch’s t-tests.** Effect sizes are reported as **Hedges’ g.** The **DERS total score reflects overall difficulties in emotion regulation.**
