# Supplementary material for: Psychological and lifestyle correlates of eating behavior and adiposity: Structural and latent profile modeling
Source: PLoS One. 2026 Feb 20;21(2):e0343336. doi: 10.1371/journal.pone.0343336 (PMC12922993; doi:10.1371/journal.pone.0343336)
Supplement: S9 File — Between-class comparisons for eating behaviors (EO, HO, DR), Unhealthy Diet Index (UDI), physical activity (IPAQ_MET), and sedentary behavior (Sitting). (DOCX) [file pone.0343336.s009.docx]

**S9 File. Behavioral validators**

**Between-class comparisons for eating behaviors, diet quality, physical activity, and sedentary behavior.**

**Table S9. Behavioral validators across latent profiles (Low-risk vs High-risk)**

| **Variable** | **Low-risk M (SD)** | **High-risk M (SD)** | **t (Welch)** | **p** | **Hedges g** |
| --- | --- | --- | --- | --- | --- |
| Emotional overeating (EO) | 4.24 (0.87) | 7.08 (1.06) | 56.77 | <.001 | 2.91 |
| Habitual overeating (HO) | 4.23 (0.86) | 7.19 (1.03) | 60.52 | <.001 | 3.11 |
| Dietary restraint (DR) | 4.22 (0.85) | 7.05 (1.07) | 56.69 | <.001 | 2.91 |
| Unhealthy Diet Index (UDI) | 3.57 (0.31) | 4.09 (0.34) | 31.47 | <.001 | 1.62 |
| Physical activity (IPAQ total MET-min/week) | 5429.39 (1505.36) | 4597.36 (1481.98) | -10.78 | <.001 | -0.56 |
| Sedentary time (IPAQ sitting) | 4.08 (0.50) | 4.51 (0.51) | 16.35 | <.001 | 0.84 |

**Note.** Low-risk profile: LPA_Class = 2 (n = 727). High-risk profile: LPA_Class = 1 (n = 773). Group differences were tested using **Welch’s t-tests**. Effect sizes are reported as **Hedges’ g.** Positive values indicate higher scores in the High-risk profile.
