# Supplementary material for: Psychological and lifestyle correlates of eating behavior and adiposity: Structural and latent profile modeling
Source: PLoS One. 2026 Feb 20;21(2):e0343336. doi: 10.1371/journal.pone.0343336 (PMC12922993; doi:10.1371/journal.pone.0343336)
Supplement: S10 File — Example R and Python code illustrating data preparation, CFA, SEM, multi-group analyses, and latent profile analysis procedures used in the study. (DOCX) [file pone.0343336.s010.docx]

**Supplementary File 10. Statistical Software and Example Code**

The code provided below is illustrative and intended to enhance transparency rather than enable exact replication.

**Software environment**

- **R (version 4.3.1):** used for structural equation modeling (SEM), moderation, and multi-group invariance tests.
  - Packages: *lavaan* (Rosseel, 2012), *semTools*, *psych*.
- **R (tidyLPA):** used for latent profile analysis (LPA) with robust estimation and multiple random starts.
- **Python (version 3.11):** used for preliminary descriptive analyses and data handling.
  - Packages: *pandas*, *numpy*, *scipy*.

**Handling missing data and outliers**

- SEM models were estimated with full information maximum likelihood (FIML).
- Outliers in continuous variables were addressed using robust estimation / winsorization at the 1st and 99th percentile.

**Bootstrapping**

- Indirect effects were tested with bias-corrected bootstrapping (5,000 resamples, 95% CI).

**Example R code: SEM (lavaan)**

library(lavaan)

library(semTools)

# Define higher-order model

model <- '

# measurement model

EMS =~ ysq1 + ysq2 + ysq3 + ... + ysq18

DERS =~ ders1 + ders2 + ders3 + ...

Support =~ mspss1 + mspss2 + mspss3 + mspss4

# structural paths with interactions

DERS ~ EMS * Stress + EMS * Support

EO ~ DERS

HO ~ DERS

DR ~ DERS

UDI ~ EO + HO + DR

BMI ~ UDI + IPAQ_MET + Sitting

WC ~ UDI + IPAQ_MET + Sitting

'

fit <- sem(model, data = dataset,

estimator = "MLR", se = "bootstrap",

bootstrap = 5000, missing = "fiml")

summary(fit, standardized = TRUE, fit.measures = TRUE, ci = TRUE)

**Example R code: Multi-group SEM**

fit_config <- sem(model, data = dataset, group = "gender", estimator = "MLR")

fit_metric <- sem(model, data = dataset, group = "gender", group.equal = "loadings")

fit_scalar <- sem(model, data = dataset, group = "gender", group.equal = c("loadings", "intercepts"))

anova(fit_config, fit_metric, fit_scalar)

**Example R code: Latent Profile Analysis (tidyLPA)**

library(tidyLPA)

# Variables standardized beforehand

lpa_results <- estimate_profiles(dataset[, c("EMS", "DERS", "Stress", "Support",

"EO", "HO", "DR", "UDI", "IPAQ_MET", "Sitting")],

1:5, model = 1, nrep = 50)

compare_solutions(lpa_results)

# Extract class membership

dataset$Class <- get_data(lpa_results[[2]])$Class

**Example Python code: Descriptive statistics**

import pandas as pd

import numpy as np

from scipy import stats

# Load dataset

df = pd.read_csv("data.csv")

# Descriptive statistics

desc = df.describe()

# Normality tests

for col in ["BMI", "WC", "EO", "HO", "DR"]:

k2, p = stats.normaltest(df[col].dropna())

print(col, "p =", p)
