# Supplementary figures and images for: Psychological and lifestyle correlates of eating behavior and adiposity: Structural and latent profile modeling
Source: PLoS One. 2026 Feb 20;21(2):e0343336. doi: 10.1371/journal.pone.0343336 (PMC12922993; doi:10.1371/journal.pone.0343336)

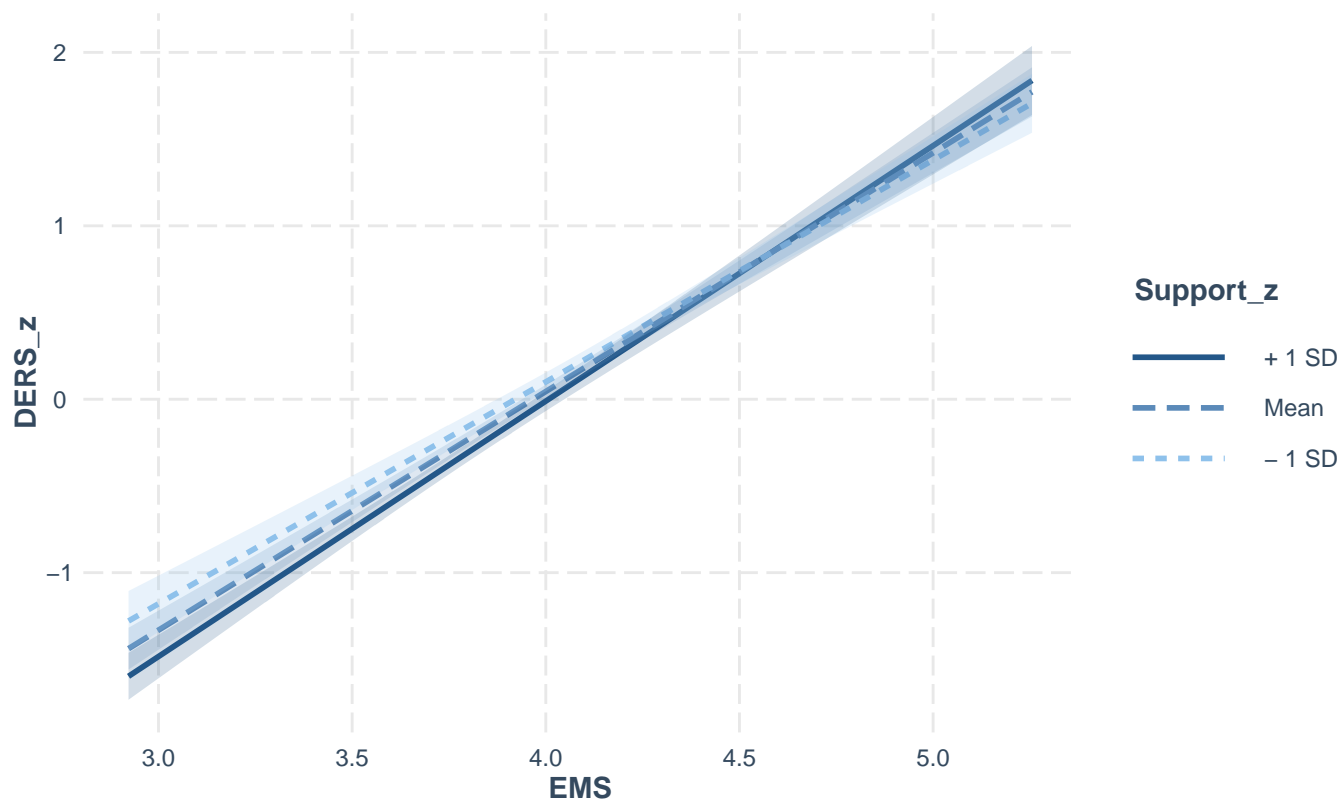

Supplement: S1 Fig — Values are standardized (z-scores). Lines represent estimated simple slopes at −1 SD (low social support), mean, and +1 SD (high social support). (PDF) [file pone.0343336.s011.pdf]
